# Fast characterization of segmental duplication structure in multiple genome assemblies

**DOI:** 10.1186/s13015-022-00210-2

**Published:** 2022-03-18

**Authors:** Hamza Išerić, Can Alkan, Faraz Hach, Ibrahim Numanagić

**Affiliations:** 1 Department of Computer Science, University of Victoria, Victoria, BC V8P 5C2 Canada; 2 Department of Computer Engineering, Bilkent University, 06800 Ankara, Turkey; 3 Vancouver Prostate Centre, Vancouver, BC V6H 3Z6 Canada; 4 Department of Urologic Sciences, University of British Columbia, Vancouver, BC V5Z 1M9 Canada

**Keywords:** Genome analysis, Fast alignment, Segmental duplications, Sequence decomposition

## Abstract

**Motivation:**

The increasing availability of high-quality genome assemblies raised interest in the characterization of genomic architecture. Major architectural elements, such as common repeats and segmental duplications (SDs), increase genome plasticity that stimulates further evolution by changing the genomic structure and inventing new genes. Optimal computation of SDs within a genome requires quadratic-time local alignment algorithms that are impractical due to the size of most genomes. Additionally, to perform evolutionary analysis, one needs to characterize SDs in multiple genomes and find relations between those SDs and unique (non-duplicated) segments in other genomes. A naïve approach consisting of multiple sequence alignment would make the optimal solution to this problem even more impractical. Thus there is a need for fast and accurate algorithms to characterize SD structure in multiple genome assemblies to better understand the evolutionary forces that shaped the genomes of today.

**Results:**

Here we introduce a new approach, BISER, to quickly detect SDs in multiple genomes and identify elementary SDs and core duplicons that drive the formation of such SDs. BISER improves earlier tools by (i) scaling the detection of SDs with low homology to multiple genomes while introducing further 7–33$\times$ speed-ups over the existing tools, and by (ii) characterizing elementary SDs and detecting core duplicons to help trace the evolutionary history of duplications to as far as 300 million years.

**Availability and implementation:**

BISER is implemented in Seq programming language and is publicly available at https://github.com/0xTCG/biser.

## Introduction

Segmental duplications (SDs), also known as low-copy repeats, are genomic segments larger than 1 Kbp that are duplicated one or more times in a given genome with a high level of homology [1]. While nearly all eukaryotic genomes harbor SDs, it is the human genome that exhibits the largest diversity of SDs among the known genomes. At least 6% of the human genome is covered by SDs ranging from 1 Kbp to a few megabases [1]. The architecture of human SDs also differs from other mammalian species both in its complexity and frequency [2]. For example, while most species harbor tandem SDs, the human genome is repleted with interspersed (both intra- and inter-chromosomal) SD blocks [3]. Human SDs are also often duplicated multiple times within the genome, often immediately next to or even within an already existing SD cluster. This complex duplication architecture points to a major role that SDs play in human evolution [4–6]. Human SDs also introduce a significant level of genomic instability that results in increased susceptibility to various diseases [7, 8]. This has led to evolutionary adaptation in the shape of genes and transcripts unique to the human genome that aim to offset the effects of such instability [9]. Finally, SDs display significant diversity across different human populations and can be used as one of the markers for population genetics studies [10].

In order to understand the architecture and evolution of eukaryotic SDs, the first step typically consists of detecting all SDs within a given genome. However, SD detection is a computationally costly problem. The theoretically optimal solution to this problem—a local alignment of an entire genome to itself—is unfeasible due to large sizes of eukaryotic genomes that render the classical quadratic time algorithms such as Smith–Waterman impractical. Furthermore, the homology levels between SD copies—as low as 75%—prevent the use of the available edit distance approximations with theoretical guarantees [11, 12]. This is likely to remain so due to the sub-quadratic inapproximability of edit distance metrics [13]. The vast majority of sequence search and whole-genome alignment tools that rely on heuristics to compute the local alignments, such as MUMmer [14] and BLAST [15], also assume high levels of identity between two sequences and therefore are not able to efficiently find evolutionarily older SD regions. Even specialized aligners for noisy long reads, such as Minimap2 [16] or MashMap [17], cannot handle 75% homology that is lower than the expected noise of long reads (up to 15%, although sequencing error rates have been improved recently to 5%) [18]. Finally, even if we use higher homology thresholds (such as 90%) to define an SD, the presence of low-complexity repeats and the complex SD rearrangement architecture often prevents the off-the-shelf use of the existing search and alignment tools for detecting SDs.

For these reasons, only a few SD detection tools have been developed in the last two decades, and most of them employ various heuristics and workarounds—often without any theoretical guarantees—to quickly find a set of acceptable SDs. The gold standard for SD detection, Whole-Genome Assembly Comparison (WGAC), uses various techniques such as hard-masking and alignment chunking to find SDs [1]. While its output is used as the canonical set of SDs in the currently available genomes, and as such, forms the basis of the vast majority of SD analysis studies, WGAC can only find recent or highly conserved SDs (i.e., those with > 90% homology) within primate lineages. Furthermore, WGAC requires specialized hardware to run and takes several days to complete. Few other tools developed as a replacement for WGAC—namely SDdetector [19] and ASGART [20]—are also limited in their ability to find older SDs with lower homology rates. Currently, the only tools that are able to detect SDs with lower homology are SDquest [21] and SEDEF [22]. SEDEF combines the unique biological properties of SD evolutionary process with Poisson error model and MinHash approximation scheme, previously used for long read alignment [17], to quickly find SDs even with 75% homology, while also providing basic theoretical guarantees about the sensitivity of the search process. SDquest, on the other hand, relies on *k*-mer counting to find putative SD regions that are later extended and aligned with LASTZ [23].

It should be noted that an SD is often formed by copying parts of older, more ancient SDs to a different location. This, in turn, implies that each SD can be decomposed into a set of short building blocks, where each block either stems from an ancient SD or a newly copied genomic region. Such building blocks are called “elementary SDs” [2]. Elementary SDs are often shared across related species within the same evolutionary branch. It has been proposed that a small subset of elementary SDs—often dubbed *seeds* or *core duplicons*—evolutionarily drives the whole SD formation process and that every SD harbors at least one such core duplicon [2]. Core duplicons are further used to hierarchically cluster SDs into distinct clades. For example, the human genome SDs can be divided into 435 duplicon blocks that are further classified into 24 clades, seeded by a set of core duplicons with a total span of 2 Mbps that is often gene-rich and transcriptionally active [2]. The prime example of a mosaic-like recombination region that is seeded by an SD core is the *LCR16* locus of the human genome that is shared with many other primates [3].

The proper SD evolutionary history analysis and the detection of core duplicons require a joint analysis of SDs in many related species. However, while existing SD tools can find SDs in single genomes in a reasonable amount of time, none of them can scale—at least not efficiently—to multiple genome assemblies. Furthermore, no publicly available tool can provide a deeper understanding of SD evolutionary architecture or find core duplicons across different species, mostly due to the computational complexity of such analysis because of the large number of existing SDs within different species. (The source code that was used for older analyses [2] is not publicly available. SDquest, on the other hand, can detect elementary SDs but only at the single genome level. Furthermore, it does not provide exact genomic coordinates of the detected elementary SDs.) For these reasons, only a small subset of previously reported core duplicons was analyzed in-depth (e.g., *LCR16* cores), and often so by manually focusing on narrow genomic regions to make the analysis tractable [3], preventing the emergence of a clearer picture of the SD evolution across different species, especially of those SDs that preclude the primate branch of the evolutionary tree.

Here we introduce BISER (**B**risk **I**nference of **S**egmental duplication **E**volutionary st**R**ucture), a new framework implemented in Seq programming language [24, 25] that is specifically developed to quickly detect SDs even at low homology levels across multiple related genomes. BISER is also able to infer elementary and core duplicons and thus enable an evolutionary analysis of all SDs in a given set of related genomes. The key conceptual advances of BISER consist of a novel linear-time algorithm that can quickly detect regions that harbor SDs in a given set of genomes and a new approach for decomposing SDs into elementary SDs. BISER can discover SDs in the human genome in 54 CPU minutes, or in 7 min on a standard 8-core desktop CPU—an 10$\times$ speed-up over SEDEF and 33$\times$ speed-up over SDquest. Further analysis of elementary SDs takes 19 min. BISER can analyze all shared SDs in seven primate genomes in roughly 16 CPU hours, translating to 2 h on a standard 8-core laptop computer. The flexibility of BISER will make it a useful tool for SD characterizations that will open doors towards a better understanding of the complex evolutionary architecture of these functionally important genomic events.

## Methods

### Preliminaries

Consider a genomic sequence $G=g_1g_2g_3 \ldots g_{|G|}$ of length |*G*| and alphabet $\Sigma =\{A, C, G, T, N\}$. Let $G_i=g_i\ldots g_{i+n-1}$ be a substring of *G* of length *n* that starts at position *i* in *G*. To simplify the notation, the length is assumed to be *n*. We will use an explicit notation $G_{i:i+n}$ for a substring of length *n* starting at position *i* when a need arises. Let $s_1\circ s_2$ represent a string concatenation of strings $s_1$ and $s_2$. The subsequence of size *k* in a sequence *s* is called *k*-mer, and the *k*-mer set *K*(*s*) of sequence *s* is the set of all subsequences of size *k* in *s*.

Segmental duplications are long, low-copy repeats generated during genome evolution over millions of years. Following such an event, different copies of a repeat get subjected to different sets of mutations, causing them to diverge from each other over time. Thus, it is necessary to introduce a similarity metric between two strings in order to detect SDs in a given genome. To that end, we use Levenshtein’s [26] *edit distance* metric $\mathcal {E}$ between two strings *s* and $s^{\prime}$ that measures the minimum number of edit operations (i.e., substitutions, insertions, and deletions at the single nucleotide level) in the alignment of *s* and $s^{\prime}$. Let $\ell$ be the length of such alignment; it is clear that $\max (|s|, |s^{\prime}|)\le \ell \le |s| + |s^{\prime}|$. We can also define an *edit error* $err(\cdot ,\cdot )$ between *s* and $s^{\prime}$ (or, in the context of this paper, an *error*) as the normalized edit distance: $err(s,s^{\prime})=\mathcal {E}(s,s^{\prime})/\ell$. Intuitively, this corresponds to the sequence divergence of *s* and $s^{\prime}$. Now we can formally define an SD as follows:

#### **Definition 1**

A segmental duplication (SD) within the error threshold $\varepsilon$ is a tuple of paralog sequences $(G_i, G_j)$ that satisfies the following criteria:

1. $$err(G_i, G_j)\le \varepsilon$$;
2. $\ell \ge 1000$, where $\ell$ is the length of the optimal alignment between $G_i$ and $G_j$ [1]; and
3. paralog sequences $G_i$ and $G_j$ can overlap at most $\varepsilon \cdot n$ bases with each other. 1

Given a set of genomes $G^1,\ldots ,G^\gamma$ and their mutual evolutionary relationships, our goal is to:

- find a set of valid SDs, $\mathcal {SD}^i$, within each $G^i$ **(SD detection)**;
- find all copies of both *s* and $s^{\prime}$ for $(s, s^{\prime}) \in \mathcal {SD}^i$ in other genomes $G^j, j \ne i$, if such copies exist **(SD cross-species conservation detection)**; and
- decompose each SD from $\mathcal {SD}=\mathcal {SD}^1\cup \cdots \cup \mathcal {SD}^\gamma$ into a set of *elementary SDs* *E*, and determine the set of core elementary SDs (defined later) that drive the formation of SDs in $\mathcal {SD}$ **(SD decomposition)**.

To that end, we developed BISER, a computational framework that is able to efficiently perform these steps, and we describe the algorithms behind it in the following sections.

For the sake of clarity, unless otherwise noted, we assume that we operate on a single genome *G*. Since SDs are by definition different from low-complexity repeats and transposons, we also assume that all genomes $G^1,\ldots ,G^\gamma$ are hard-masked and do not contain low-complexity regions. Nearly all tools, with the sole exception of SEDEF, impose this constraint as well. The hard-masked genome can be obtained on the fly from a standard genome assembly by filtering bases represented with the lowercase bases (that correspond to low-complexity regions).

#### SD error model

Different paralogs of an SD are mutated independently of each other. Therefore, the sequence similarity of paralogs is correlated with the age of the duplication event—more recent copies are nearly identical, while distant ancestral copies are dissimilar. It has been proposed that the sequence similarity of older SDs (e.g., those shared by the mouse and the human genomes) falls as low as 75% [22]. In other words, the dissimilarity between different copies of an old SDs exceeds 25% (i.e., $err(s,s^{\prime}) \ge 0.25$ for SD paralogs *s* and $s^{\prime}$, according to the definition above).

Detection of duplicated regions within such a large error threshold is a challenging problem, as nearly any edit distance approximation technique with or without theoretical guarantees breaks down at such high levels of dissimilarity [11, 17], provided that this error is truly random. However, that is not the case: it has been previously shown [22] that the SD mutation process is an amalgamation of two independent mutation processes, namely the background point mutations (also known as *paralogous sequence variants*, or PSVs) and the large-scale block edits. As such, the overall error rate $\varepsilon$ can be expressed as a sum of two independent error rates, $\varepsilon _P$ (PSV mutation rate) and $\varepsilon _B$ (block edit rate), where only $\varepsilon _P$ is driven by a truly random mutation process.

In the case when paralogs share the 75% sequence identity, it has been shown that the random point mutations (PSVs) contribute at most 15% ($\varepsilon _P \le 0.15$) towards the total error $\varepsilon$ [22] (this also holds for many other mammalian genomes, as their substitution rate is often lower than the human substitution rate [27]). The remaining 10%—knowing that $\varepsilon _P$ and $\varepsilon _B$ are additive—is assumed to correspond to the block edit rate $\varepsilon _B$. Note that these mutations are clustered *block* errors and, as such, are not randomly distributed across SD regions. The probability of a large block event is roughly 0.5% based on the analysis of existing SD calls [22].

On the other hand, we assume that PSVs between two SD paralogs *s* and $s^{\prime}$ follow a Poisson error model [17, 28] and that those mutations occur independently from each other. It follows that any *k*-mer in $s^{\prime}$ has accumulated on average $k\cdot \varepsilon _P$ mutations compared to the originating *k*-mer in *s*, provided that such *k*-mer was part of the original copy event. By setting a Poisson parameter $\lambda = k\cdot \varepsilon _P$, we obtain the probability of a duplication event in which a *k*-mer is preserved in both SD paralogs (i.e., that a *k*-mer is error-free) to be $e^{-k \varepsilon _P}$.

### Putative SD detection

Let us return to the main problem of determining whether two strings *s* and $s^{\prime}$ are “similar enough” to be classified as SDs. As mentioned before, classical edit distance calculation algorithms would be too slow for this purpose. Instead, we use an indirect approach that measures the similarity of strings *s* and $s^{\prime}$ by counting the number of shared *k*-mers in their respective *k*-mer sets $\mathbf {K}(s)$ and $\mathbf {K}(s^{\prime})$. It has been shown that Jaccard index of these sequences, *s* and $s^{\prime}$, defined as $\mathcal {J}(\mathbf {K}(s),\mathbf {K}(s^{\prime}))=\frac{|\mathbf {K}(s)\cap \mathbf {K}(s^{\prime})|}{|\mathbf {K}(s)\cup \mathbf {K}(s^{\prime})|}$ is a good proxy for $\mathcal {E}(s,s^{\prime})$ under the Poisson error model [17]. Thus we can combine the Poisson error model with the SD error model and obtain the expected value of Jaccard index $\tau$ between any two strings *s* and $s^{\prime}$, whose edit error $err(s,s^{\prime})$ follows the SD error model and is lower than $\varepsilon = \varepsilon _P + \varepsilon _B$, to be [22]:

1 $$\tau = \mathbb {E}[\mathcal {J}(\mathbf {K}(s),\mathbf {K}(s^{\prime}))] \ge \frac{1-\varepsilon _B}{1+\varepsilon _B} \cdot \frac{1}{2e^{k\varepsilon _P}-1}.$$

As we cannot use local alignment to efficiently enumerate all SDs in a given genome due to quadratic time and space complexity, we utilize a heuristic approach to enumerate all pairs of regions in *G* that are likely to harbor one or more segmental duplications. We call these pairs *putative SDs*. These pairs are not guaranteed to contain a “true” SD, and must be later aligned to each other to ascertain the presence of true SDs. Nevertheless, such an approach will *filter out* the regions that do not harbor SDs, and thus significantly reduce the amount of work needed for detecting “true” SDs. The overall performance of our method, both in terms of runtime and sensitivity, will depend on how well the putative SDs are chosen.

The problem of putative SD detection can be, thanks to the SD error model, easily expressed as an instance of a filtering problem: find all pairs of indices *i*, *j* in *G* such that $\mathcal {J}(\mathbf {K}(G_i), \mathbf {K}(G_j)) \ge \tau$, where $\tau$ is the lower bound from the Eq. 1. Here we assume that the size of $G_i$ and $G_j$ exceeds the SD length threshold (1000 bp), and no *k*-mer occurs twice in either $G_i$ or $G_j$.^2^

The filtering approach has already been successfully used in other software packages and forms the backbone of both SEDEF (SD detection tool; [22]) and MashMap (Nanopore read aligner; [29]). However, both methods need to constantly maintain the *k*-mer sets $\mathbf {K}(s)$ and $\mathbf {K}(s^{\prime})$ to calculate the Jaccard index between the sequences *s* and $s^{\prime}$. As these methods dynamically grow *s* and $s^{\prime}$ (as the length *n* is not known in advance), the corresponding sets $\mathbf {K}(s)$ and $\mathbf {K}(s^{\prime})$ are constantly being updated, necessitating a costly recalculation of $\mathbf {K}(s)\cap \mathbf {K}(s^{\prime})$ on each update. A common trick is to use the MinHash technique to reduce the sizes of $\mathbf {K}(s)$ and $\mathbf {K}(s^{\prime})$, and thus the frequency of such updates. However, the frequent recalculation of the Jaccard index still remains a major bottleneck even in the MinHash-based approaches because calculating union and intersection of *k*-mers for each pair of subsequences in *G* is a costly operation.

**Fig. 1:**
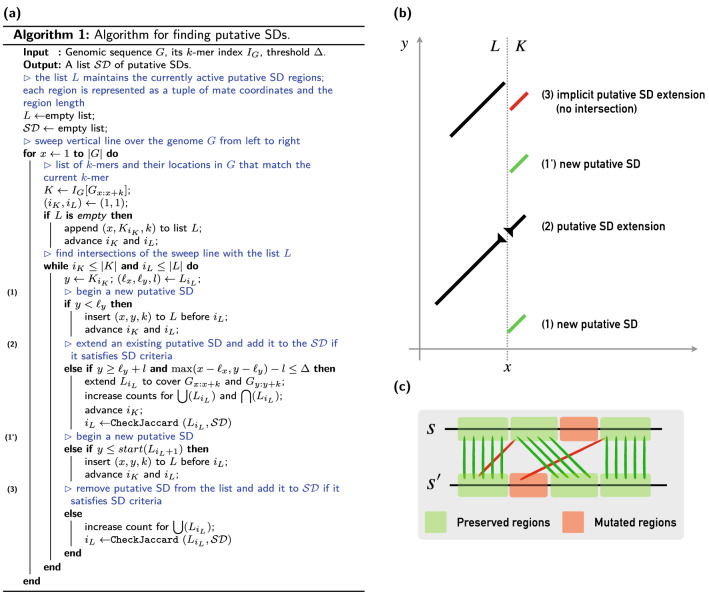
**a** A plane-sweep algorithm for finding putative SDs. **b** Visual guide for the algorithm. The algorithm sweeps a vertical dashed line through the set of winnowed *k*-mers in a genome *G* (*x* axis). At each *k*-mer starting at the location *x*, it queries the index $I_G$ to obtain a sorted list *K* of *k*’s occurrences in *G* (right side of the sweep line). The algorithm then scans *K*, and the list *L* of putative SDs found thus far at the same time. At each step, it examines $i_L$-th element of *L* and $i_K$-th element of *K*, and decides whether to start a new putative SD [(1) and (1′), green *k*-mers on the right], extend the current putative SD with the current *k*-mer [(2), black *k*-mer on the right], or subsume the current *k*-mer within the current putative SD [(3), red *k*-mer]. **c** A visual representation of a valid *k*-mer matching in a valid alignment (shown by green lines). Red matching would render the alignment invalid as red matchings are not co-linear with the green matchings

Here we note that the Jaccard index calculation can be significantly simplified by not having to maintain the complete *k*-mer sets $\mathbf {K}(s)$ and $\mathbf {K}(s^{\prime})$. The need for keeping such sets arises from the fact that the calculation of $\mathbf {K}(s)\cap \mathbf {K}(s^{\prime})$ allows any *k*-mer in $\mathbf {K}(s^{\prime})$ to match any *k*-mer in $\mathbf {K}(s)$. However, such a loose intersection requirement is not only redundant for approximation of edit distance under the SD error model but is even undesirable as such intersections can introduce cross-over *k*-mer matches that are not possible in the edit distance metric space (see Fig. 1c for an example of valid and invalid matchings). By disallowing such cross-over cases, we can significantly optimize the calculation of the Jaccard index. Let us show how to do that without sacrificing sensitivity.

Let us first introduce $s \circledast s^{\prime}$ as an alternative way of measuring the *k*-mer similarity between strings *s* and $s^{\prime}$.

For that purpose, let us introduce a notion of a *colinear* *k-mer matching* between *s* and $s^{\prime}$ as a set of index pairs (*i*, *j*) ($1\le i\le |s|,$ $1\le j\le |s^{\prime}|$) such that the *k*-mers that start at *i* and *j* in *s* and $s^{\prime}$ respectively are equal, and such that all pairs (*i*, *j*) in matching are colinear (i.e., for each (*i*, *j*) and $(i^{\prime}, j^{\prime})$, either $i<i^{\prime}$ and $j<j^{\prime}$, or $i>i^{\prime}$ and $j>j^{\prime}$). A $\circledast$ operation describes the size of a maximum colinear matching of *k*-mers between *s* and $s^{\prime}$. In other words, we want to select a maximal set of matching *k*-mers in $\mathbf {K}(s)$ and $\mathbf {K}(s^{\prime})$ such that no two *k*-mer matchings cross over each other (see Fig. 1c for an example of cross-over, or non-colinear, matchings). We can replace $\mathbf {K}(s)\cap \mathbf {K}(s^{\prime})$ with $s \circledast s^{\prime}$ and introduce an *ordered Jaccard index* $\hat{\mathcal {J}}(s,s^{\prime})$, formally defined as:

$$\hat{\mathcal {J}}(s,s^{\prime}) = \frac{s \circledast s^{\prime}}{|\mathbf {K}(s)\cup \mathbf {K}(s^{\prime})|}.$$

The following lemma allows us to use an ordered Jaccard index $\hat{\mathcal {J}}$ in lieu of classical Jaccard index $\mathcal {J}$:

#### **Lemma 1**

*Let s and* $s^{\prime}$ *be two paralog sequences that have been mutated under the assumptions of SD error model following the originating copy event. Also, assume that their shared k-mers were also shared before any mutation occurred. Then the ordered Jaccard index* $\hat{\mathcal {J}}(s, s^{\prime})$ *of s and* $s^{\prime}$ *is equal to the Jaccard index* $\mathcal {J}(\mathbf {K}(s),\mathbf {K}(s^{\prime})).$

#### *Proof*

It is sufficient to prove that the size of $|\mathbf {K}(s)\cap \mathbf {K}(s^{\prime})|$ always corresponds to the size of maximal colinear matching between *s* and $s^{\prime}$.

To show that $s \circledast s^{\prime} \le |\mathbf {K}(s)\cap \mathbf {K}(s^{\prime})|$, it is enough to note that matched *k*-mers in any colinear matching are by definition identical, and thus belong to $\mathbf {K}(s)\cap \mathbf {K}(s^{\prime})$. We will prove that $s \circledast s^{\prime} \ge |\mathbf {K}(s)\cap \mathbf {K}(s^{\prime})|$ by contradiction. First, note that the string *s* is equal to $s^{\prime}$ immediately after the duplication event (i.e., before the occurrence of PSVs) and that all *k*-mers are colinear in their maximal matching because *s* contains no repeated *k*-mers (an assumption made by the SD error model). Now, suppose that there is a cross-over in $\mathbf {K}(s)\cap \mathbf {K}(s^{\prime})$. That implies either a cross-over between *s* and $s^{\prime}$ before PSVs occurred—contradicting the previous observation—or a cross-over after it, contradicting the assumption that any matched *k*-mer pair was matched before the occurrence of PSVs. Hence $\mathbf {K}(s)\cap \mathbf {K}(s^{\prime})$ cannot contain any cross-overs, and $s \circledast s^{\prime} = |\mathbf {K}(s)\cap \mathbf {K}(s^{\prime})|$. $\square$

If the conditions of Lemma 1 are satisfied, we can calculate $s \circledast s^{\prime}$ in linear time by a simple scan through *s* and $s^{\prime}$ at the same time. A linear calculation of $s \circledast s^{\prime}$, together with the fact that the lower bound $\tau$ in Eq. 1 equally holds for $\hat{\mathcal {J}}$ as well (a direct consequence of Lemma 1), allows us to use a plane sweep technique to select all pairs of substrings $(s, s^{\prime})$ in *G* whose ordered Jaccard distance $\hat{\mathcal {J}}(s, s^{\prime})$ exceeds $\tau$, and as a result, select all putative SDs in *G* (see Fig. 1 for details).

We begin by creating a *k*-mer index $I_G$ that connects each *k*-mer in *G* to an ordered list of its respective locations in *G*. Then we sweep a vertical line in *G* from left to right while maintaining a sorted list *L* of putative SDs found thus far. For each location *x* in *G* encountered by a sweep line, we query $I_G$ to obtain a sorted list *K* containing loci of $G_{x:x+k}$’s copies in *G*. Then, for any *y* in *K*, we check if it either (1) begins a new potential putative SD that maps *x* to *y*, (2) extends an existing putative SD, or (3) is covered by existing putative SD in *L* (Fig. 1). If a putative SD in *L* is too distant from *y*, it is promoted to the final list of putative SD regions if it satisfies the ordered Jaccard index threshold $\tau$ and the other SD criteria from Definition 1. Note that we do not allow a *k*-mer to extend a putative SD if the distance between it and the SD exceeds the maximum gap size of the smallest possible SD (250). It takes $|L| + |K|$ steps to process each *k*-mer in *G* because both *L* and *K* are sorted. However, because the size of |*L*| is kept low by the distance criteria, and because |*K*| is low enough in practice^3^, the practical time complexity of Algorithm 1 (Fig. 1) is *O*(|*G*|) (theoretically, the worst-case complexity is $O((|L|+|K|)\cdot |G|)$) for constructing the index $I_G$, and linear in terms of the genome size for plane sweeping.

The key assumption in Lemma 1—that two paralogs only share the *k*-mers that have not been mutated since the copy event—does not always hold in practice on real data. As such, Algorithm 1 (Fig. 1) might occasionally underestimate the value of $\hat{\mathcal {J}}$, potentially leading to some false negatives. We control that by using $\Delta$—the same parameter that controls the growth of putative SDs by limiting the maximum distance of neighboring *k*-mers in $s \circledast s^{\prime}$ (Fig. 1)—to limit the growth of under-estimated SDs and thus start the growth of potentially more successful SDs earlier. This heuristic might cause a large SD to be reported as a set of smaller disjoint SD regions. For that reason, we post-process the set of putative SDs upon the completion of Algorithm 1 (Fig. 1) and merge any two SDs that are close to each other if their union satisfies the ordered Jaccard index criteria. We also extend each putative SD by 5 Kbp both upstream and downstream to account for the small SD regions that might have been filtered out during the search process. This parameter is user-defined and might be adjusted for different genome assemblies.

The performance of the plane sweep technique can be further improved by winnowing the set of *k*-mers used for the construction of $I_G$ [17]. Instead of indexing all *k*-mers in *G*, we only consider *k*-mers in a *winnowing fingerprint W*(*G*) of *G*. *W*(*G*) is calculated by sliding a window of size *w* through *G* and by taking in each window a lexicographically smallest *k*-mer (the rightmost *k*-mer is selected in case of a tie).

The expected size of *W*(*G*) for a random sequence *G* is $2|G|/(w+1)$ [30]. The main benefit of winnowing is that it can significantly speed up the search step (up to an order of magnitude) without sacrificing sensitivity. The winnow *W*(*G*) can be computed in a streaming fashion in linear time using *O*(*w*) space with the appropriate data structures (deque) [31].

Following the discovery of putative SDs, we locally align paralogs from each putative SD and only keep those regions whose size satisfies the SD criteria mentioned above. BISER uses a two-tiered local chaining algorithm from SEDEF based on a seed-and-extend approach and efficient $O(n \log n)$ chaining method following by a SIMD-parallelized sparse dynamic programming algorithm to calculate the boundaries of the final SD regions and their alignments [16, 32, 33].

### SD decomposition

**Fig. 2:**
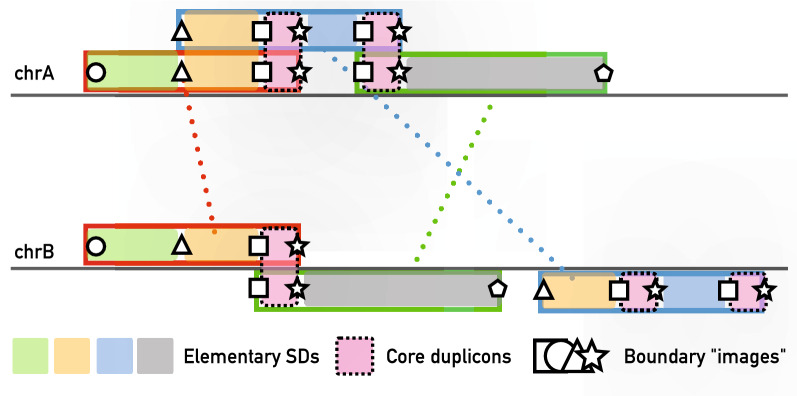
A decomposition of three partially overlapping SDs into a set of elementary SDs. Each SD is bounded by a solid line box. SD paralogs are linked by a by a dashed line. Each elementary SD is represented as a colored box. The boxes of core duplicons—elementary SDs shared by all SDs—are depicted with a dashed border. Note that a boundary of each elementary SD either a boundary of an existing SD or its “image”. Different boundaries are represented by different shapes, and their “images” (paralog copies) also share the same shape

Once the set of final SDs $\mathcal {SD}=\{(s_1, s^{\prime}_1), \ldots \}$ is discovered and the precise global alignment of each paralog pair $(s, s^{\prime}) \in \mathcal {SD}$ is calculated, we proceed by decomposing the set $\mathcal {SD}$ into a set of evolutionary building blocks called *elementary SDs*. More formally, we aim to find a minimal set of elementary SDs $E=\{e_1,\ldots ,e_{|E|}\}$, such that each SD paralog *s* is a concatenation of $\hat{e}^s_1 \circ \cdots \circ \hat{e}^s_{n_s}$. Each $\hat{e}_i$ either belongs to *E* or there is some $e_j \in E$ such that $err(\hat{e}_i, e_j) \le \varepsilon$. An example of such a decomposition is given in Fig. 2.

**Fig. 3:**
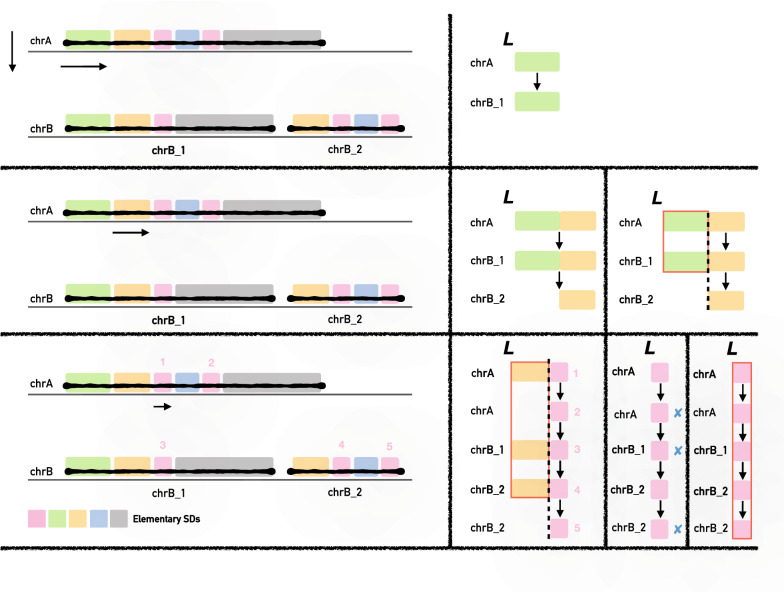
*k*-mer chaining-based SD decomposition applied on the example from Fig. 2. Top: after data pre-processing, we end up with three sequences (*chrA*, *chrB_1*, and *chrB_2*) that are scanned from left to right to find identical regions that share common *k*-mers. The first matching region is the green region in *chrA* that matches the same-colored region in *chrB_1*. Middle: after encountering the yellow region (b), the algorithm marks a new elementary SD because the number of yellow regions does not match the number of green regions; therefore, the green regions will be reported as instances of a separate elementary SD. Bottom: if no *k*-mer can be appended to any of the elementary SDs in *L*, the algorithm will report all regions that are larger than $\mu$ as one elementary SD and discard the others. Here, the regions numbered as 2, 3, and 5 do not continue into the blue regions and thus prevent the further extension of the pink region

Note that each locus covered by an SD paralog is either copied to another locus during the formation of that SD (in other words, it is “mirrored” by its paralog), or belongs to an alignment gap. As SD events can copy over the regions that already form an existing SD, a single locus might “mirror” a large number of existing locations. In order to find all locations that a locus *i* mirrors, we initially used a modification of Tarjan’s union-find disjoint set algorithm [34] to link together all mirrored locations. Each separate “mirror” (represented by a distinct shape in Fig. 2) indicates the start of a distinct elementary SD. However, despite being efficient and conceptually simple, the simple version of this algorithm cannot handle the complex SD alignments that often induce mirror loops, whirls, bulges stemming from the alignment imperfections [21, 35]. These artifacts prevent the formation of larger elementary SDs that can be meaningfully analyzed. The current solutions to this problem—most notably the A-Bruijn graph family of repeat analysis tools [2, 35, 36]—is limited to small genomes and unfortunately not scale well to large datasets (Fig. 3).

For that reason, we developed an alternative approach to decompose SDs into elementary SDs motivated by the fact that the SD decomposition is closely related to the multiple sequence alignment problem. We start by denoting the set of all regions in genome *G* that contains SDs as *R*. By definition, separate instances of the same elementary SDs are supposed to be similar and therefore should consist of identical *k*-mers that can be chained. We define *chaining* as the merging of proximal locations of identical *k*-mers. The chaining process resembles the local multiple sequence alignment on *R*, and produces a set of duplicated regions in *R*. Two *k*-mers can be chained if their locations are within defined parameter $d_g$. Parameter $d_g$ has two purposes: (1) it defines the maximum distance up to which one *k*-mer location can be merged with another, and (2) it ensures that there will be at least one matching *k*-mer every $d_g$ locations in each LMSA, thus reducing the number of false positives and random hits. We found out that the optimal value of $d_g$ is 50 if the goal is to cover elementary SDs of size 100 and larger [2]. Such $d_g$ is large enough to capture regions that contain PSVs and small gaps, but small enough to prevent false positives.

The decomposition step itself is modeled upon Algorithm 1 (Fig. 1; decomposition is described in Fig. 3) and proceeds as follows. We build a *k*-mer index $I_k$ of *R* as explained above (except that this time we do not use winnowed *k*-mers). Then we scan all sequences using the same sweeping line algorithm as before. The list *L* keeps putative elementary SDs found so far. Whenever we process a new *k*-mer, we will take all locations from $I_k$ and see if we can: (1) append them to an existing putative elementary SD from *L* (if *L* is empty, we initialize it with the current *k*-mer’s positions); (2) create a new potential elementary SD; or (3) remove an existing one if it satisfies the deletion criteria. A new location from $I_k$ can be appended to an existing elementary SD if its distance from the last appended *k*-mer to that elementary SD is within $d_g$. A putative elementary SD is removed if no new *k*-mer location is appended to that putative elementary SD in $d_g$ steps. The main difference from the putative SD search step is that we need to track multiple copies of a putative region instead of only one (because an elementary SD can belong to multiple SDs). For this purpose, when removing a node from *L*, we also need to remove all other nodes from *L* to form an elementary SD set (if such node is larger than the threshold $\mu$).

The computational performance of this approach heavily depends on the size of an $I_k$. To reduce its size, we cluster all overlapping SDs, merge sequences that overlap, and apply the same algorithm on every cluster separately in parallel, reducing each cluster’s index size. Clustering SDs is done using Tarjan’s union-find algorithm [34]. The largest cluster for human SDs covers roughly 90 megabases, meaning that those SDs exhibit a rich evolutionary history that can be tracked by breaking those SDs into elementary SDs.

After decomposing SDs into the set of elementary SDs *E*, we select some of them as *core duplicons*. Inspired by [2], we formally define these duplicons as the minimal set of elementary SDs that cover all existing SDs (an SD is covered by an elementary if either paralog is composed of that elementary SD). We use a classical set-cover approximation algorithm [37] to determine a set of core duplicons from *E*.

### Multiple genomes

The above method can be efficiently scaled to $\gamma$ distinct genomes $G^1,\ldots ,G^\gamma$ by constructing a set of *k*-mer indices $I_{G^1}, \ldots , I_{G^\gamma }$, and by running the search and the alignment procedure on each $G^i$ in parallel. After obtaining SDs for each genome $G^1,\ldots ,G^\gamma$ in parallel, BISER maps the set of SDs of a genome to all other genomes. By only mapping the SDs of one genome to another genome, BISER avoids misclassifying conserved regions between two genomes as SDs. The whole procedure can be trivially parallelized across many CPUs.

## Results

We have evaluated all stages of BISER for speed and accuracy on both simulated and real-data datasets. All results were obtained on a multi-core Intel Xeon 8260 CPU (2.40 GHz) machine with 1 TB of RAM. The run times are rounded to the nearest minute and are reported for both single-core as well as multi-core (8 CPU cores) modes when ran in parallel via GNU Parallel [38]. All real-data genomes were hard-masked, and all basepair coverage statistics are provided with respect to the hard-masked genomes.

In our experiments, we used $k=14$ when searching for putative SDs and $k=10$ during the alignment step (note that both parameters are user-adjustable). The size of the winnowing window was set to 16. The lower values of *k* significantly impact the running time without providing any visible improvement to the detection sensitivity, while higher values of *k* significantly lower the detection sensitivity. The genome decomposition step used $k=10$. Both *k* and *w* (for search, align, and *k*-mer chaining decomposition) were empirically chosen to maximize sensitivity without impacting the runtime performance. Parameter selection details and sensitivity analysis are available in [39].

### Simulations

**Fig. 4:**
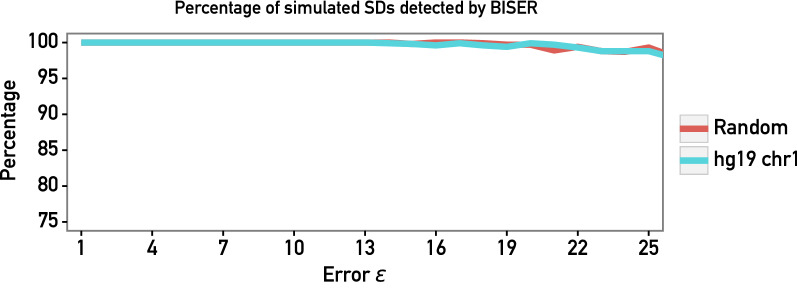
Performance of BISER’s algorithm on simulated SDs (red: randomly simulated sequences; cyan: hg19 chr1 sequences). *x*-Axis represents the simulated SD error rate $\varepsilon$, while *y* axis represents the percentage of correctly detected SDs. Note that the *y*-axis only shows the top 25% as BISER detects more than 98% of simulated SDs for any $\varepsilon \le 0.25$

The accuracy of using the strong Jaccard index together with the SD error model as a function of error parameter $\varepsilon$, as well as the overall sensitivity of BISER’s SD detection pipeline, was evaluated on a set of 1,000 simulated segmental duplications ranging from 1 to 100Kbp. All sequences and mutations were randomly generated with uniform distribution according to the SD error model with $\varepsilon \in \{0.01,0.02, \ldots ,0.25\}$ (i.e., we allowed the overall error rate to reach 25%). Uniform distribution was picked because it was an overall good biological proxy for mutation in known genomes and because it can represent worst-case mutation distribution (having one mutation on each non-overlapped *k*-mer). We consider a simulated SD as being “covered” if BISER found an SD that covers more than 90% of the original SD’s basepairs. As shown in Fig. 4, the overall sensitivity is around 99% even for $\varepsilon = 0.25$.

We performed the same experiment on human (hg19) chromosome 1 (Fig. 4), where we selected uniformly at random 10,000 sequences of various lengths and duplicated them within the chromosome. Each duplication was followed by introducing random PSVs according to the SD error model while varying the values of $\varepsilon$ as described above. Even in this case, BISER’s performance stays the same, and only a handful of very small SDs (of size $\approx$ 1000) were missed.

### Single-genome results

We have run BISER on the *H. sapiens* hg19 genome and *M. musculus* mm8 genome and compared it to the published WGAC [1],^4^ SEDEF [22], and SDquest [21] SD calls.^5^ We also compared the runtime performance of BISER to that of SEDEF and SDquest. Note that we were not able to run WGAC due to the lack of hardware necessary for its execution. We did not compare BISER to other SD detection tools—namely SDdetector [19], MashMap2 [29], and ASGART [20]—as it has been previously shown that these tools underperform when compared to SEDEF or SDquest, and require an order of magnitude more resources than either SEDEF or SDquest do. For the same reason, we did not compare BISER to whole-genome aligners such as Minimap2 [16] and MUMmer/nucmer [14], as well as DupMasker [40], as none of these tools were designed to detect *de novo* SDs in a genome. See [22] for the detailed evaluation of these tools.

BISER was able to find and align all SD regions in hg19 in 7 min on 8 cores (roughly 54 min on a single core) (Table 1). To put this into perspective, BISER is around 10$\times$ faster than SEDEF, 34$\times$ faster than SDquest, and an order of magnitude faster than WGAC that takes days to find human SDs (personal communication; we were not able to run the WGAC pipeline ourselves due to legacy hardware requirements). As a side note, BISER has the same memory requirements as SEDEF or SDquest and needs around 7 GB of RAM per core (it needs around 2 GB for the search step and up to 7 GB for the sequence alignment).

**Table 1:** Running time performance of BISER (single-core and 8-core mode) on Intel Xeon 8260 CPU at 2.40 GHz for single genomes (hg19 and mm8)

Single genome (hg19)	Total (min)	Putative SDs (min)	Alignment (min)
1 core	54	21	33
8 cores	7	3	4

Single genome (mm8)	Total	Putative SDs (min)	Alignment (min)
1 core	1 h 24 min	20	64
8 cores	11 min	3	9

Since SEDEF by default operates on a genome that is not hard-masked, we also ran SEDEF on a hard-masked genome to measure its theoretical speed (note that SEDEF was not designed for hard-masked genomes; thus, the basepair analysis is omitted). SEDEF took 21 min on 8 CPU cores to process a hard-masked hg19, leaving it still around 3$\times$ slower than BISER. Noticeable speedup is obtained in the first step of the algorithm—finding putative SDs—where SEDEF completes in 14 min while BISER needs only 3 min.

Similar performance gains were observed on the mouse (mm8) genome as well. BISER took 11 min to find SDs in the mm8 genome (3 min for finding putative SDs and 9 min for alignment) while SEDEF needed 1 h and 24 min (33 min for finding putative SDs and 51 min for align). SDquest took more than 6 h for the same operation. SEDEF was run on soft masked data; when we ran it on hard masked data, it took 27 min. Here, the speedup is shown in the first step—finding putative SDs—where BISER needs 3 min compared to the SEDEF’s 18 min.

**Fig. 5:**
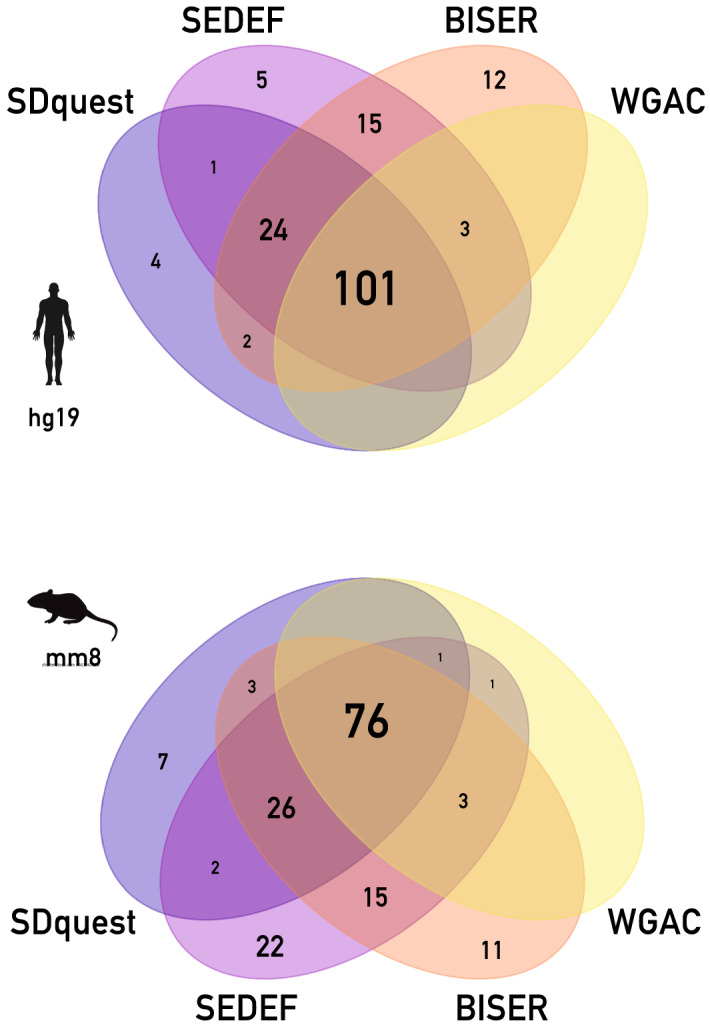
Venn diagram depicts the SD coverage of the BISER, WGAC, SEDEF and SDquest (in Mbp) on the hard-masked human genome (top) and mouse genome (bottom). Note that nearly all bases out of $\approx$ 22 Mbp bases that are shown to be unique to SEDEF (and not covered by BISER) map to gaps and low-copy repeats and should not be therefore treated as true SDs

In terms of sensitivity, BISER discovers about 1 GB of putative SD regions in the human genome. After the alignment step, BISER reports 158 Mb of final SD regions in hg19. That is 54 Mbp more than WGAC and 26 Mbp more than SDquest. The total coverage of SEDEF and BISER are similar to each other, differing by 4 Mbp uniquely detected by SEDEF and 12 Mbp uniquely covered by BISER. BISER also misses a few Mbp of SD regions unique to SDquest and a negligible amount unique to WGAC (Fig. 5 and Table 3).

On the mm8 genome, we observe similar trends. However, we also observed that SEDEF covers roughly 20 Mbp that are not covered by BISER (Fig. 5 and Table 3). When SEDEF is run on a hard-masked genome, it does not cover these bases; further analysis showed that nearly all bases originally reported as unique to SEDEF actually map either to alignment gaps, soft-masked repeat elements, or small islands (< 200 bp) between the low-copy repeats and as such do not constitute “true” SDs.

### Decomposition

The BISER’s decomposition module found 297,175 elementary SDs grouped in 65,222 elementary SD sets. The method covers 85% of the SD basepairs. The minimum length of an elementary SD was set to 100 bp. BISER needs roughly 20 min on 8 cores to perform the single-genome decomposition (19 min for hg19 and 18 min for mm8).

**Fig. 6:**
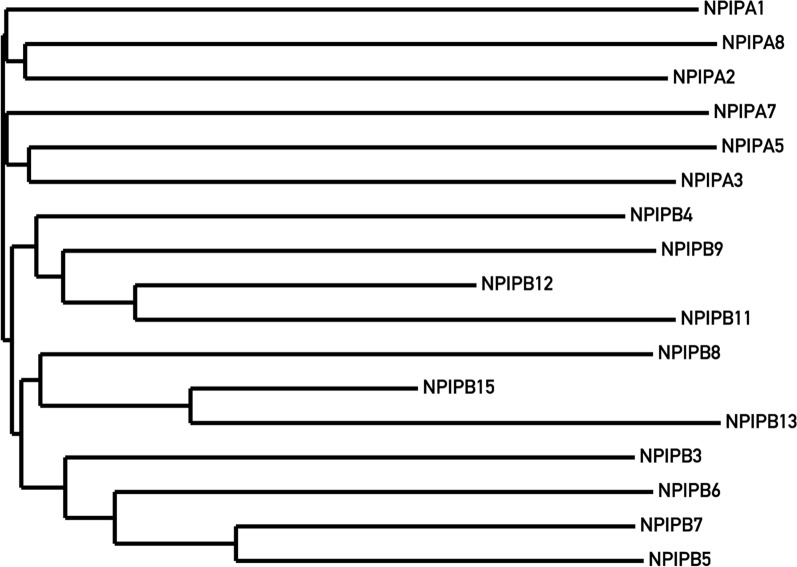
A phylogenetic tree of *NPIP* gene family built by using elementary SD similarity as the proximity metrics (neighbor-joining method)

To validate the results of decomposition, we performed the phylogenetic analysis of the prominent *NPIP* gene cluster from the LCR16 region in the human genome, and compared our results with the previously published analysis of this region [3]. Distances between genes were calculated as $d(s_1,s_2) = 1 - \mathcal {J}(s_1, s_2)$, where $\mathcal {J}$ is Jaccard similarity between two sets of elementary SDs covering two respective genes (as each genic region is covered by one or more elementary SDs). As can be seen in Fig. 6, BISER’s correctly inferred the evolutionary tree for this gene family, as the generated tree agrees with the one previously reported in [3].

While SDquest produces (for one genome) SDs and mosaic SDs composed of indexes of elementary SDs, those indexes do not give us the information on the exact coordinates of each elementary SD needed for tree reconstruction. For that reason, we were not able to compare our results with to SDquest.

### Multi-genome results

In addition to running BISER on a single genome, we also ran BISER on the following seven related genomes:

- *M. musculus* (mouse, version mm8),
- *C. jacchus* (marmoset, version calJac3),
- *M. mulatta* (macaque, version rheMac10),
- *G. gorilla* (gorilla, version gorGor6),
- *P. abelii* (orangutan, version ponAbe3),
- *P. troglodytes* (chimpanzee, version panTro6), and
- *H. sapiens* (human, version hg19).

These genomes were analyzed in the previous work [3], with the sole exception of *M. musculus* that is novel to this analysis.

BISER took around 2 h to complete the run on 8 cores. Of that, it took around 35 min to find putative SDs within and between species. The remaining time (1 h 32 min) was spent calculating the final alignments for all reported SDs (Table 2). The vast majority of alignment time was spent only on aligning putative SDs from calJac3 genome. We presume that this is due to the high presence of unmasked low-complexity regions in this particular assembly.

**Table 2:** Running time performance of BISER (single-core and 8-core mode) on Intel Xeon 8260 CPU at 2.40 GHz for seven genomes

Seven genomes	Total	Putative SDs	Alignment
1 core	16 h 41 min	4 h 37 min	12 h 4 min
8 cores	2 h 7 min	35 min	1 h 32 min

The SD decomposition took 37 min on 8 CPU cores (67 min on a single CPU) to complete on a set of nearly 1,985,586 SDs. BISER found $\approx$ 282,130 elementary SDs (Table 3).

**Table 3:** SD coverage of the human and mouse genomes (hg19 and mm8) and the runtime performance of BISER, SEDEF and SDquest

Tool	Covered (MBp)	Missed (MBp)	Extra (MBp)	Time
**hg19**				
WGAC (standard)	104.5			days
BISER	158.0	0.5	54.0	7 min
SEDEF	149.2	0.1	44.8	1 h 15 min
SDquest	132.2	3.3	30.9	3 h 56 min
**mm8**				
WGAC (standard)	80.3			days
BISER	135.1	1.3	56.0	11 min
SEDEF	145.6	0.2	65.4	1 h 24 min
SDquest	115.7	3.8	39.2	6 h 06 min

“Missed” and “Extra” columns are calculated with respect to the WGAC SD calls. All running times are reported on 8 CPU cores. We could not run WGAC as we do not have access to the legacy hardware needed for its execution; the reported runtime is from  [22]

## Conclusion

More than a decade ago, the Genome 10K Project Consortium proposed to build genome assemblies for 10,000 species [41]. Due to the lack of high-quality long-read sequencing data, this aim was not immediately realized. However, the Genome 10K Project spearheaded the development of other large-scale many-genome sequencing projects such as the Earth BioGenome Project [42] and Vertebrate Genomes Project.^6^ Recent developments in generating more accurate long-read sequencing data, coupled with better algorithms to assemble genomes now promise to make the aforementioned and similar projects feasible.

Analyzing the recently and soon-to-be generated genome assemblies to understand evolution requires the development of various algorithms for different purposes, from gene annotation [43] to orthology analysis [44] and the selection and recombination analysis [45]. Although a handful of tools such as SEDEF and SDquest are now available to characterize segmental duplications in genome assemblies, they cannot perform multi-species SD analysis, and they suffer from computational requirements. We developed BISER as a new segmental duplication characterization algorithm to be added to the arsenal of evolution analysis tools. We demonstrate that (1) BISER is substantially faster than earlier tools; (2) it can characterize SDs in multiple genomes to delineate the evolutionary history of duplications; and (3) it can identify elementary SDs and core duplicons to help understand the mechanisms that give rise to SDs. We believe that BISER will be a powerful and common tool and will contribute to our understanding of SD evolution when thousands of genome assemblies become available in the next few years. The following steps would consist of applying BISER to a larger set of available mammalian genomes, and the detailed biological analysis of the SDs and associated core duplicons.

## Footnotes

1 Ideally, the SD mates should not overlap; however, due to the presence of errors, we need to account for at most $\varepsilon \cdot n$ overlap.

2 Even if it does, the above-derived Jaccard score-based filter performs well in practice.

3 The average size of *L* in our experiments was 370, and the average size of *K* is 30.

4 http://humanparalogy.gs.washington.edu

5 The exact coverage depends on search parameters, such as the minimum putative SD length (set to 500 and 100 bp for the query and the reference sequence, respectively). Other parameter choices do not significantly affect the final result: extra false positives are quickly filtered by the align step due to a lack of shared regions that need to be chained.

6 https://vertebrategenomesproject.org/.
